# Stainless-Steel Antenna on Conductive Substrate for an SHM Sensor System with High Power Demand

**DOI:** 10.3390/s21237841

**Published:** 2021-11-25

**Authors:** Sarah Bornemann, Jan Niklas Haus, Michael Sinapius, Björn Lüssem, Andreas Dietzel, Walter Lang

**Affiliations:** 1 Institute for Microsensors, Actuators and Systems, University of Bremen, 28359 Bremen, Germany; bluessem@imsas.uni-bremen.de (B.L.); wlang@imsas.uni-bremen.de (W.L.); 2 Institute of Microtechnology, Technische Universität Braunschweig, 38124 Braunschweig, Germany; j.haus@tu-braunschweig.de (J.N.H.); a.dietzel@tu-braunschweig.de (A.D.); 3 Institute of Mechanics and Adaptronics, Technische Universität Braunschweig, 38106 Braunschweig, Germany; m.sinapius@tu-braunschweig.de

**Keywords:** structural health monitoring, fiber metal laminates, antenna, energy harvesting, ferrite, stainless steel, SHM, FML

## Abstract

This paper presents the novel concept of structuring a planar coil antenna structured into the outermost stainless-steel layer of a fiber metal laminate (FML) and investigating its performance. Furthermore, the antenna is modified to sufficiently work on inhomogeneous conductive substrates such as carbon-fiber-reinforced polymers (CFRP) independent from their application-dependent layer configuration, since the influence on antenna performance was expected to be configuration-dependent. The effects of different stack-ups on antenna characteristics and strategies to cope with these influences are investigated. The purpose was to create a wireless self-sustained sensor node for an embedded structural health monitoring (SHM) system inside the monitored material itself. The requirements of such a system are investigated, and measurements on the amount of wireless power that can be harvested are conducted. Mechanical investigations are performed to identify the antenna shape that produces the least wound to the material, and electrical investigations are executed to prove the on-conductor optimization concept. Furthermore, a suitable process to fabricate such antennas is introduced. First measurements fulfilled the expectations: the measured antenna structure prototype could provide up to 11 mW to a sensor node inside the FML component.

## 1. Introduction

In recent years, fiber compounds have increasingly been used in lightweight construction, which has led to increased research efforts on manufacturing processes, material improvement, and the development of structural health monitoring (SHM) systems. Often, fiber-reinforced plastic and thin metal layers are combined to form fiber metal laminates (FMLs), which have the benefits of both materials for application. In particular, the glass laminate aluminum reinforced epoxy (GLARE) has attracted attention as it is used as fuselage material for the Airbus A380, but other material combinations also provide promising properties.

The use of FMLs in safety critical applications leads to increased efforts to predict or detect damages early on. Numerous methods for structural health monitoring, i.e., to detect damage inside composite structures, have been proposed. The work of Diamanti et al. [1] provided a detailed review on these methods.

Although several external approaches exist, an integrated SHM system that can easily be read by a single handheld device would present a major advancement, as it immensely reduces the time and effort for maintenance. Therefore, a sensor node must be embedded into the FML by hybrid integration [2] without disturbing the composite structure [3,4]. Such a system must either be powered by an internal energy source such as a battery or be independent from battery lifetime by an external source via an antenna. Furthermore, data provided by the sensor node can be transmitted via a wireless interface to a reader device.

Passive radio frequency identification (RFID) technology is suited for this application, as energy harvested from the reader field can power the sensor node inside the material while data are simultaneously transferred. Therefore, the embedded sensor node is represented as the transponder part of the RFID system, i.e., commercially available readers can be used to power the sensor node and read out sensor data.

This article proposes a novel concept for the integration of the antenna into the FML by structuring its outer metal layer. Methods to decouple the antenna from the underlying, potentially inhomogeneous, conductive fiber compound material are discussed. The mechanical influence of the antenna on the load-bearing material layer could be minimized by optimizing the antenna geometry.

Different concepts for RFID antennas on conductive substrates were investigated [5,6]. Most of these approaches could not be used here, the coil could not be wound on a ferrite core, and the antenna could not be separated from the conductive material by a larger gap. However, the approach proposed by Ohmura [7] showed that a sintered ferrite sheet beneath the antenna coil area achieved good performance for a transponder working close to a metal layer.

This is the starting point for the following investigations. However, the carbon-fiber-reinforced polymer (CFRP) layers used here were more complex than metal layers used by Ohmura. In particular, the orientation of the carbon fibers inside the composite material influenced the strength of coupling to the antenna, complicating analysis in comparison to isotropic materials such as metals. Here, a thin layer of a ferromagnetic material directly beneath the RFID antenna structure redirected the electromagnetic field, which decoupled the antenna from the substrate and allowed for optimizing the antenna independent of the particular configuration of the FML.

### Project Context

**Figure 1:**
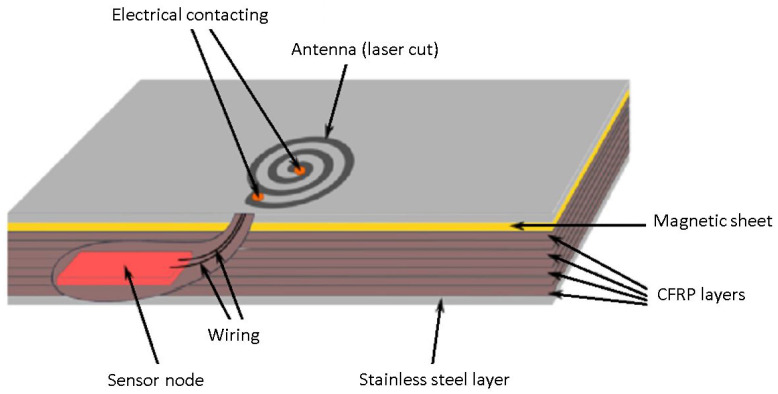
Concept of FML SHM system with laser-structured antenna.

The proposed antenna was cut by lasers into the outer steel layer of an FML structure consisting of CFRP and stainless-steel layers. The sensor electronics were embedded inside the material stack. Figure 1 shows a schematic overview of the concept.

The passive RFID-based sensor node approach used here had the advantage of a minimized construction space and maximized lifetime, since a large energy storage, e.g., a battery, was not needed for operation.

However, the conductive CFRP material used as a substrate material complicated the design of the antenna [8,9], and the remaining stainless steel around the antenna structure itself. In addition, the electrical conductivity and thereby electromagnetic characteristics of CFRP strongly depend on fiber content, fiber orientation, and layer configuration [10,11].

High-permeability layers, also known as ferrite sheets, inserted between conductive materials and RFID antennas reduce detuning and attenuation effects [12]. The reader device, necessary for energy and data transfer, induces a field that leads to eddy currents inside the conductive material and causes the problems mentioned before. The penetration of the magnetic fields of the reader device cannot be completely prevented, but the field can be redirected to a sufficient amount through this additional layer to minimize this effect.

Apart from providing the data interface for sensor measurements, the antenna must be able to provide enough energy for the whole sensor node to function properly during data collection. Therefore, the power consumption of the planned electronic system was estimated, and the energy-harvesting capability of the system was measured to show that it is possible to realize sufficient energy and data transfer at RFID standardized 13.56 MHz.

The promising results of the measurements carried out for this paper are provided in Section 3. The negative effects of conductive CFRP could be neglected if the approach of inserting a ferrite layer into the material stack is followed. This additional layer provides the advantage of electrical insulation between the antenna structure and the carbon fibers inside the CFRP. Furthermore, the antenna is able to harvest a sufficient amount of energy from the externally provided reader field, even with a nonoptimized reader antenna, to completely provide the estimated required energy for the sensor system.

## 2. Materials and Methods

**Figure 2:**
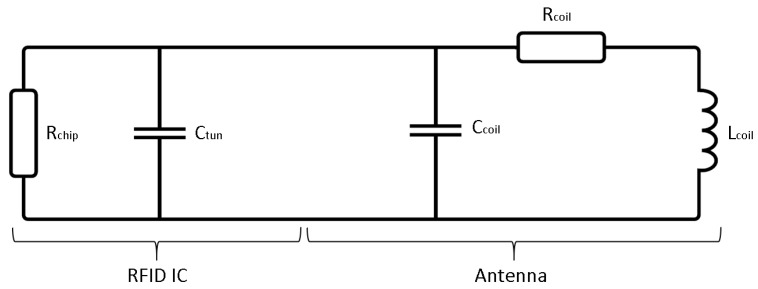
Equivalent circuit diagram of passive RFID tag.

The design of planar spiral coils for high-frequency RFID antennas is well-known, at least for antennas used in nonconductive environments. Figure 2 shows an equivalent circuit that represents the combination of antenna and the RFID integrated circuit (IC), which were combined into a working RFID tag.

Manufacturers of RFID ICs deliver the information on the range of internal tuning capacity $C_{tun}$ of their devices, and often also provide a guide on antenna design strategies. Some manufacturers have even designed supporting software to specify the parameters of the antenna and obtain the calculated inductance value.

### 2.1. Basic Equations and Design Parameters

There are several approaches that can be used to approximate the inductance of an antenna. Wheeler’s equation [13] is the basis for recommended approximation Equation (1) for spiral coils provided in the application note on printed antenna design from ST Microelectronics.

(1) $$L_{coil}=31.33\;\cdot \;\mu_{0}\;\cdot \;N^{2}\;\cdot \;\frac{{\left(\frac{r_{in}+r_{out}}{2}\right)}^{2}}{8\;\cdot \;\frac{r_{in}+r_{out}}{2}+11\;\cdot \;\left(r_{out}-r_{in}\right)}$$

where $r_{in}$, inner radius in m; $r_{out}$, outer radius in m; $L_{coil}$, antenna coil inductance in Henry; and N, number of windings. The outer radius is often defined by the particular application, for example, the maximal outer dimensions of a credit card. In this work, $r_{out}$ was not directly restricted to a fixed number, but was chosen to be as small as possible to also keep the material wound as small as possible. $r_{out}$ was thus chosen to be in the range of common commercially available tags, about the length of the short side of a credit card, with the possibility to increase the size if the amount of harvested energy was not sufficient for the SHM sensor node.

Tuning capacity $C_{tun}$ was determined by the RFID IC used in the design. As Figure 2 shows, it is in parallel to parasitic capacitance $C_{coil}$, which could be assumed to be much smaller than the tuning capacity, and could thereby be neglected for first design calculations. The influence of $C_{coil}$ was taken into account later on in the design procedure, when three models of the antenna ($L_{coil}$, $L_{coil}$ − 5%, $L_{coil}$ + 5%) were produced to choose the one showing the best performance with a workflow recommended by the RFID IC manufacturer. The influence of $R_{coil}$ was also not considered in these calculations, as it influences the quality factor of the produced antenna but not the resonance frequency of the design. The required inductance value for a given tuning capacity $C_{tun}$ and targeted resonance frequency $f_{res}$ could be calculated using Equation (2).

(2) $$L_{coil}=\frac{1}{4\pi^{2}\;\cdot \;f_{res}^{2}\;\cdot \;C_{tun}}$$

This gives a theoretical value of 4.92 μH for a resonance frequency of 13.56 MHz using the tag in air. Equation (1) can be converted into representing the resulting inner radius depending on a given inductance and outer radius, which were the design-dependent parameters.

(3) $$r_{in}=2\;\cdot \;\sqrt{\frac{22\;\cdot \;L_{coil}}{31.33\;\cdot \;\mu_{0}\;\cdot \;N^{2}}\;\cdot \;r_{out}+{\left(\frac{7\;\cdot \;L_{coil}}{31.33\;\cdot \;\mu_{0}\;\cdot \;N^{2}}\right)}^{2}}-r_{out}-\frac{14\;\cdot \;L_{coil}}{31.33\;\cdot \;\mu_{0}\;\cdot \;N^{2}}$$

Equation (3) was the basis for the design of the presented stainless-steel antennas and was used to obtain the initial design parameters depending on the required inductance value of the antenna. Due to the later design additions (surrounding stainless steel and ferrite sheet beneath), the obtained and calculated inductance values differed, and the latter was adjusted in an iterative design circle.

### 2.2. CAD Design

**Figure 3:**
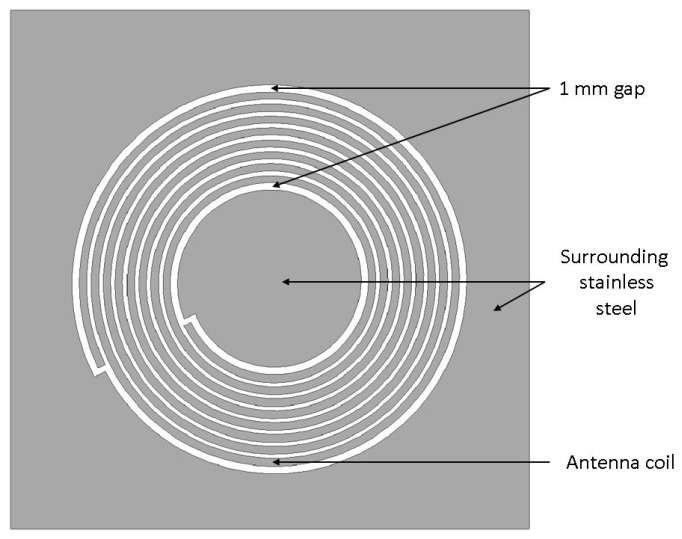
Antenna structure (top view) with stainless-steel areas surrounding coil.

The geometry of the antenna was designed in CAD software using the determined parameters as described above. The remaining areas of stainless steel within and around the antenna structure itself were not included in the above calculations. Due to this approach, the resulting shift in resonance frequency due to parasitic capacitances was compensated by iterative changes on the antenna geometry. A top view of the antenna design is presented in Figure 3. A 1 mm gap was included around the antenna to reduce the parasitic capacity effects of the surrounding material on the coil as far as possible, as this was the maximal gap taken from project requirements.

### 2.3. Manufacturing

**Figure 4:**
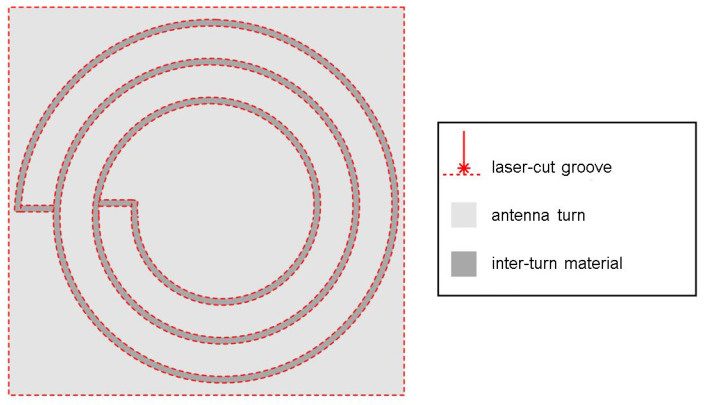
Antenna structuring process by FS laser ablation.

Femtosecond laser ablation was used to cut contours into a 100 μm thick stainless spring steel sheet (DIN EN 10088 1.4310). A laser micromachining workstation (microSTRUCT C by 3D Micromac), equipped with a Yb:KGW femtosecond laser source (PHAROS by Light Conversion) was used, which emits at λ= 1028 nm. For fast patterning, the laser beam was deflected using a scanner (Intelliscan 14 by Scanlab GmbH) and a f=100 mm telecentric FTheta Lens (Linos AG). Per antenna turn, two contour grooves were laser-cut into the sheet metal as shown in Figure 4 using the parameters listed in Table 1.

The number of repetitions was optimized, so that a minimally thin residual metal layer remained and mechanically stabilized the flatness of the antenna geometry. If the turns were completely released, mechanical stresses in the material would eventually deform the antenna. Then, heat-resistant adhesive tape was manually applied onto the antenna surface to hold the antenna turns in place. Consecutively, the interturn material was removed by manually lifting it up using tweezers and a little out-of-plane force.

For the first measurements, the antenna structure remained on the adhesive tape applied to it. For later applications, the idea was to transport the structure with this tape, electrically contact it, and then establish an adhesive bond with the ferrite sheet and insert the stack into an exposed CFRP region of the FML. After this process, the adhesive tape could be removed.

### 2.4. Contacting

Two different methods were tested to permanently contact the ends of the stainless-steel antenna structures. Soldering to stainless steel did not work well, presumably because of the relatively large surface of the coil combined with good temperature conduction properties. Very good results were obtained by using electrically conductive glue (Elecolit 327 by Panacol). For antenna handling, a thin coverage with UV hardening glue is recommended to increase the connections’ mechanical stability. For later in-material-contacting, the UV glue step is not necessary.

### 2.5. Layer Fixation Setup

**Figure 5:**
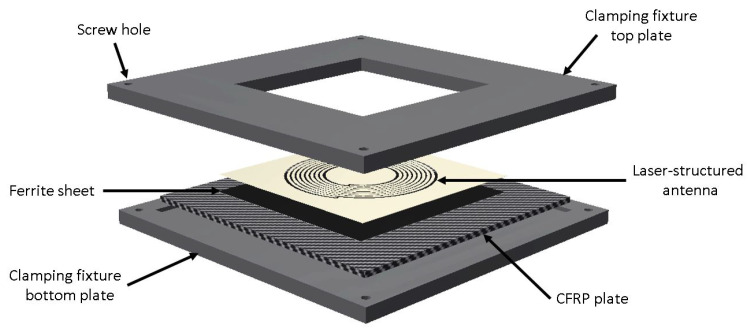
Schematic representation of clamping fixture.

Since the antenna structure was initially fabricated without the underlying magnetic foils and CFRP layers, a fixture depicted in Figure 5 was designed to attach the antenna as close as possible to the other components at this stage for reproducible results. This fixture contains a small recess into which different CFRP plates of the same geometry could be inserted in exactly the desired orientation and position. Another recess inside the top plate was used to fixate the laser-structured antenna. The ferrite sheet was glued onto one side of every sample CFRP plate to allow for measuring on ferrite and directly on CFRP by turning the plate around. The whole stack of the antenna, ferrite, and CFRP could be compressed to rebuild the later material stack, using this fixture and applying four screws and tightening them with nuts.

**Figure A1:**
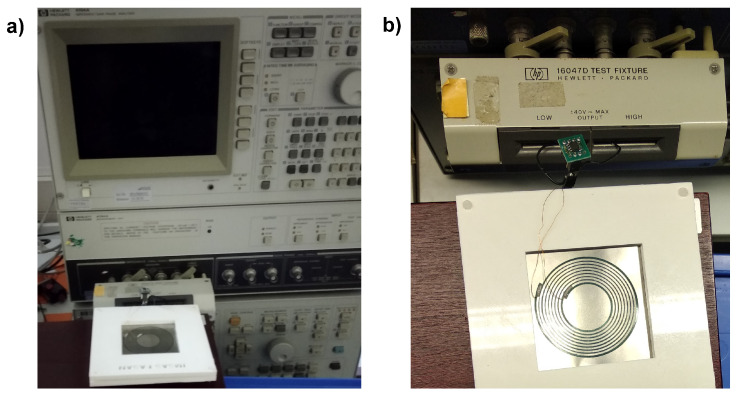
Measurement setup for impedance over frequency measurement. (**a**) Measurement setup with used impedance analyzer; (**b**) close up view on tested device.

**Figure A2:**
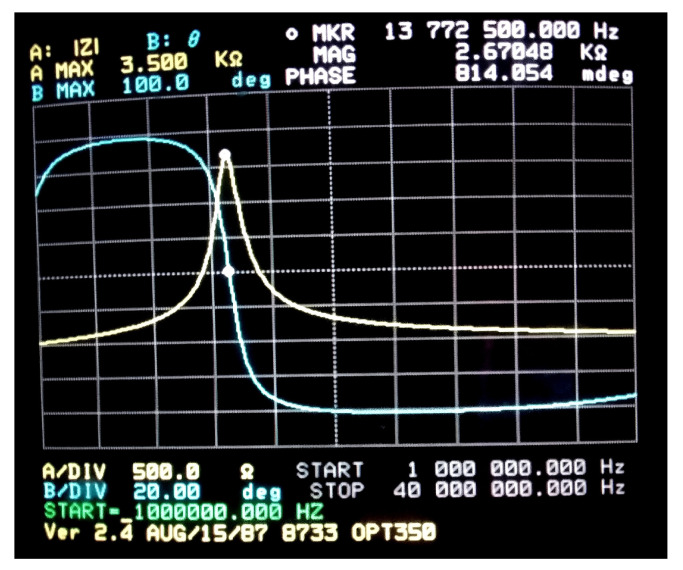
Photograph of impedance analyzer screen during measurement.

For impedance measurements, an impedance analyzer (HP 4194A) combined with a clamping fixture (16047D) was used. The instrument was calibrated using its internal CAL function for OPEN and SHORT with the attached clamping device and the contacting wires leading to the antenna. During OPEN calibration, connecting wires must be placed similar to their positions during measurement to consider their additional parasitic capacitance when placed close to each other. A photograph of the measurement setup is shown in Figure A1 and a photograph of the instrument’s screen during measurements is depicted in Figure A2, both in Appendix A.

### 2.6. Layer Orientation of CFRP Plates

**Figure 6:**
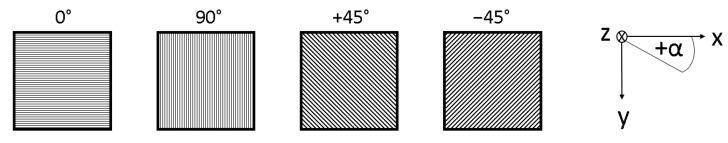
Schematic representation of the prepreg layers inside CFRP test plates. Fiber orientations represented by black lines.

The CFRP plates used here were chosen to represent a selection of different CFRP configurations that may occur beneath the antenna in dependence of the particular application of the FML. The chosen configuration mainly depends on the desired mechanical properties of the FML component that is produced. If loads on the components are expected to mainly act in one direction, a unidirectional approach is advantageous. For most applications, however, a multidirectional layer configuration is chosen because expected loads are also multidirectional. Because various layer configurations are possible, three different layer stacks were chosen to investigate possible differences in the influence on an RFID antenna’s performance. The layer orientation definition is shown in Figure 6.

Table 2 lists the different layer configurations for the used plates. The following names are used in the upcoming sections to refer to these plate specimen:

- Unidirectional CFRP—UD
- Multidirectional CFRP 0${}^{\circ }$/45${}^{\circ }$—MD45
- Multidirectional CFRP 0${}^{\circ }$/90${}^{\circ }$—MD90

## 3. Results and Discussion

As a first step of our investigation, the process of the geometry selection by a mechanical FEM simulation is explained. After that, we present impedance measurements to determine the resonance frequency of the antenna and the antenna-ferrite-CFRP system in different configurations, and design adaptations due to application-specific requirements and their effects. Lastly, the power requirement of the sensor node to realize the SHM system inside the material is estimated, and the maximal energy-harvesting capabilities of the designed antenna structure connected to the RFID IC using the IC ’s harvesting feature are validated.

### 3.1. Mechanical Simulation of Antenna Shape

**Figure 7:**
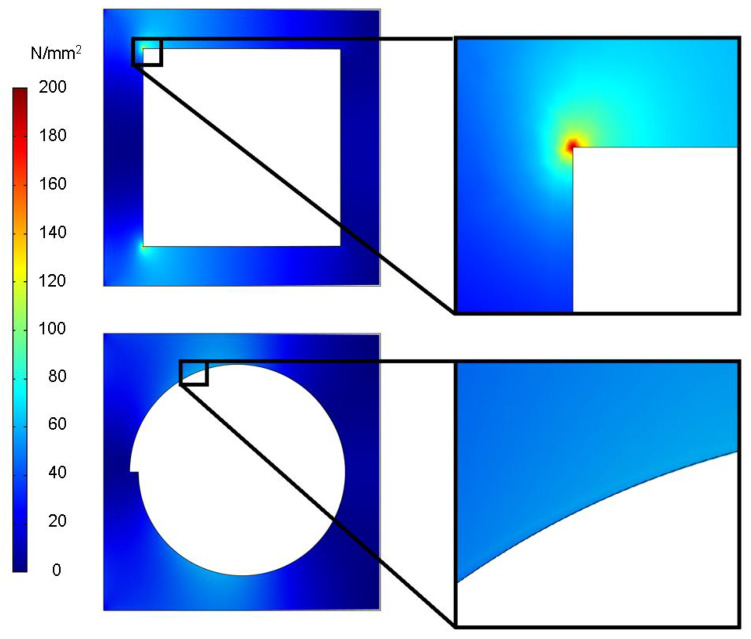
Stress caused by voids in material for different antenna shapes.

The antenna is intended to be structured into the outer material layer of the FML in the final application, so that there is a void inside the material in which the antenna structure is placed. An FEM simulation was performed investigating the impact of different geometric voids inside a 120 μm thick, 70 mm by 70 mm stainless-steel plate. The dimensions were chosen to comply with the plate size and steel foil thickness of the targeted prototype. This step investigates the influence of different void shapes on the stress distribution for one stainless-steel layer without CFRP layers beneath, since only the top layer contained a hole in the shape of the antenna. Therefore, the plate was first simulated without voids to obtain the maximal von Mises stress over the plate without an opening in the material. Plates with rectangular and circular voids were then modeled. The plate was fixed onto the left side and bent down on the opposite side. Von Mises stress is depicted for the investigation. The 0.2% proof strength for the investigated type of stainless-steel 1.4310 (X10CrNi18-8) is provided with 195 N/m$m^{-2}$ in the material data sheet. Figure 7 depicts the comparison between the two different void shapes.

The simulated force on the right edge of the plate was iteratively increased to 52 mN pressing downwards on the plate’s edge in the z direction until the rectangular void showed a peak stress slightly above the given 0.2% proof strength with 199 N/m$m^{-2}$. The stress was evenly distributed for the circular shape, while the rectangular shape led to significant stress peaks. Applying the same load to the plate’s edge with the circular void reduced the peak stress on the structure to 60.8 N/m$m^{-2}$ to only 30.55% of that of the rectangular void.

On the basis of these results, a circular antenna geometry was selected to avoid structural damage to the metal surface. In general, when aiming for minimal material weakening, a circular geometry is expected to always be superior. Furthermore, the performance of rectangular antennas, especially if their length-to-width ratio is much different than 1, is expected to depend on their position relative to the carbon fibers inside the CFRP. Circular antennas are expected to not show this placement-dependent behavior.

### 3.2. Manufactured Specimen

Table 3 lists the parameters of the antenna design based on the simulation results. The newly designed specimen after initial tests of the influence of CFRP and ferrite plates under the antenna is depicted in Figure 3. Results presented in the following sections are based on this antenna design. The design parameters listed in Table 3 show that the antenna inductance was reduced to 3.01 μH, shifting the resonance frequency of the RFID system in air to a theoretical value of 17.18 MHz. This design choice was due to an iterative design adaptation by measuring the influence of the combined components antenna, ferrite sheet, and CFRP plates. The shift in frequency strongly depends on the permeability of the ferrite layer and slightly on its thickness. For the ferrite layer, EM15TF-012-1 from 3M was chosen due to its small thickness compared to that of other commercially available products.

**Figure 8:**
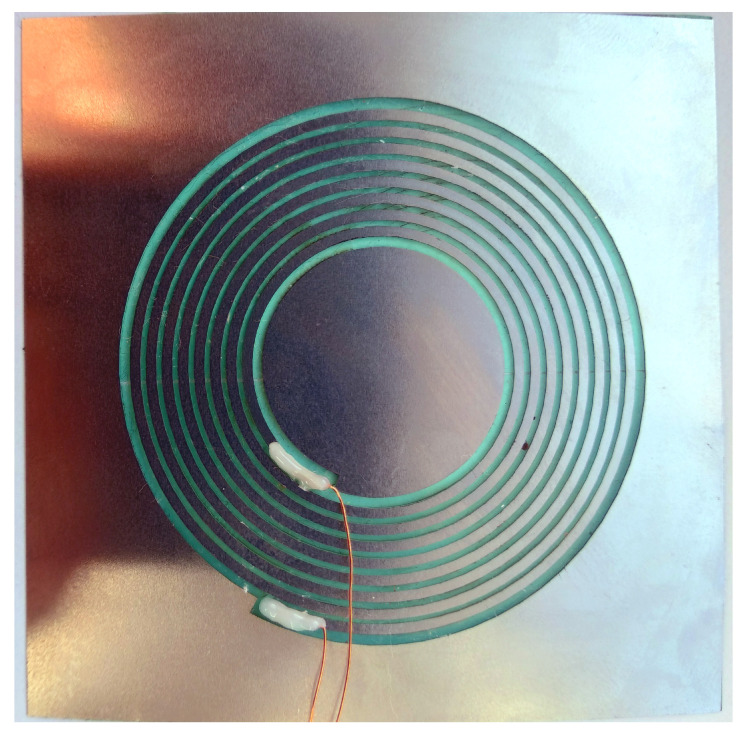
Manufactured antenna produces by FS laser ablation into a contacted stainless-steel plate.

The antenna produced using the FS laser ablation technique as described above can be seen in Figure 8. The void that was required inside the stainless steel plate that was simulated inside the mechanical simulation in the previous chapter is clearly visible. The previously described contact is also depicted.

### 3.3. Impedance Analyzer Measurements

The resonant frequency of the resulting RFID system was verified by impedance spectroscopy from 1 to 40 MHz. The resonance frequency of the antenna itself without the tuning capacity of the RFID IC (ST25DV64K) could not be identified within this range, but was expected to be close to 45 MHz, which would result into a stray capacity between the windings of the antenna of less than 4.16 pF. Analytical approximations confirmed this as a realistic value.

**Figure 9:**
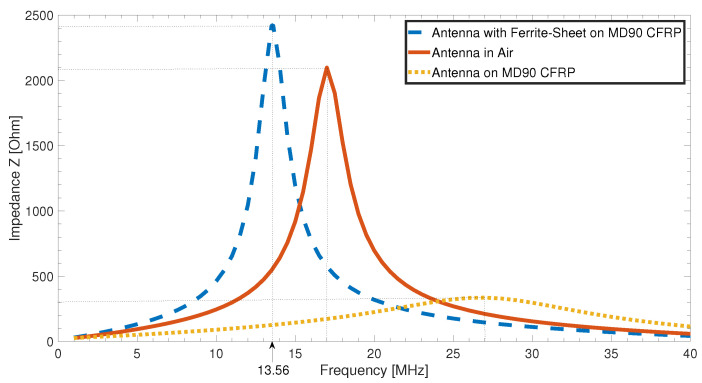
Impedance measurements of tag in air (red, solid), on CFRP (yellow, dotted), and combined with ferrite sheet and CFRP (blue, dashed).

Figure 9 shows the impedances of the antenna measured on different substrates. The solid red curve represents the impedance of the antenna on its adhesive tape placed in the air without magnetically influencing materials near it. The resonance frequency was 17 MHz, which is quite close to the theoretically calculated resonance frequency of 17.18 MHz. The quality factor, determined by the maximal impedance value divided by the bandwidth of the impedance at −3 dB, is Q=5. An almost direct placement on a CFRP plate with a 0${}^{\circ }$/90${}^{\circ }$ layer configuration with only the magnetically transparent handling sheet inbetween leads to a shift of the resonance frequency to 27 MHz, shown by the yellow dotted curve. Additionally to the frequency shift, the impedance curve is also strongly attenuated, the quality factor reduces to Q=1.38. These two effects combined are the reason why the RFID system would not work close to this CFRP plate without further additions.

One measure to allow for the system to work on a CFRP substrate is to place a ferrite sheet inbetween. The dashed blue curve in Figure 9 shows the result. There was even a slight increase in impedance compared to the antenna in air, and the quality factor improved to Q=5.23. Additionally, the resonance frequency was reduced to the desired value of 13.6 MHz, which is the optimal measurement result for a high-frequency RFID tag.

**Figure 10:**
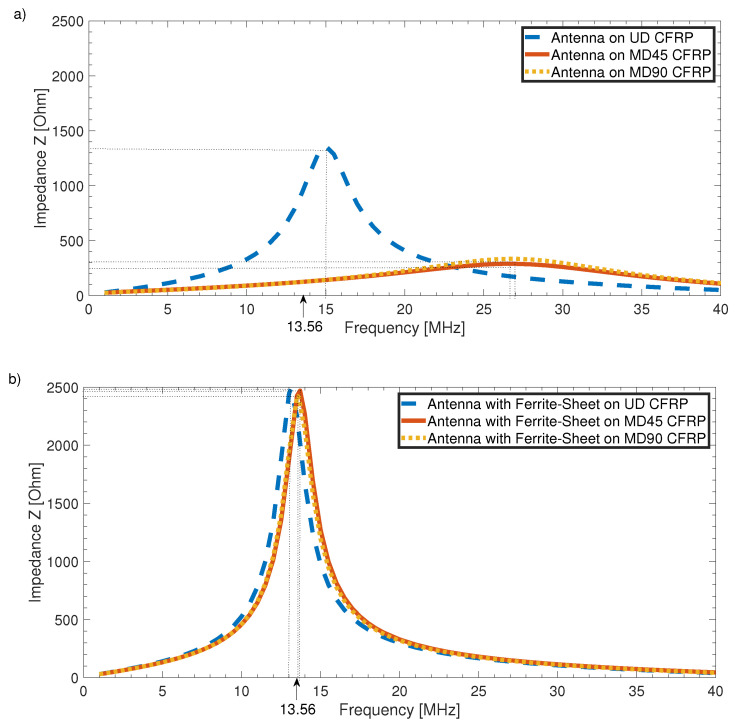
(**a**) Frequency shift for different CFRP configurations as substrate. (**b**) Resonance frequency for antennas on ferrite sheet on different CFRP configurations as substrate.

As the frequency shift due to CFRP and ferrite under the antenna can be taken into account by changing the inductance value of the antenna geometry, for direct application on CFRP, it might be possible to shift the frequency back from 27 to 13.6 MHz. Theoretically, this might work, but Figure 10a shows that the frequency shift strongly depends on the configuration of the CFRP substrate plate. The dotted yellow and solid red lines showed almost no difference in frequency shift and attenuation; both were CFRP stacks with alternating fiber directions and thus comparably conductive in the z direction. The dashed blue curve, on the other hand, was a plate of unidirectional fiber layers where the resonance frequency had only been shifted to 15 MHz, with attenuation also being significantly lower. Considering this difference, we could also assume that different fiber volume content affects antenna performance and the manufacturing process of each single CFRP component, which theoretically contains more or less contacted fibers. Moreover, the influence of the conductivity of carbon fibers decreases the quality factor of the resonating circuit to Q=2.83 for the unidirectional case, and to Q=1.38 (yellow dotted line) or even Q=1.22 (solid red line) for the multidirectional CFRP substrates. With such low-quality factors, the range of the system would be very low even without any shift of resonance frequency, which prevents high-frequency RFID communication in the first place.

Figure 10b shows the obvious advantages of introducing a thin layer of ferrite into the material stack: the independence of the substrate materials’ configuration, a retuning of resonance frequencies, and a large increase in quality factor. For all configurations, the final resonance frequency only slightly differed. This amount was within the tolerances set by the RFID standard. While reaching the desired resonance frequency, the quality factor increased and became independent from the used substrate. The quality factor for all three configurations was Q=4.7, which was an improvement of up to 285% compared to the worst quality factor directly on CFRP. Another advantage of using a ferrite layer under the antenna coil is the electrical insulation provided by the ferrite, which would otherwise be necessary to avoid shorts in the antenna windings.

### 3.4. Energy-Harvesting Capabilities of the System

The antenna structure not only enables data transfer from the sensor system to a reading unit, but also provides the sensor system with a sufficient amount of power for operation. Table 4 lists an estimate of the power demand for the embedded electronic system. On the basis of this estimation, the system requires 5.7 mW to supply the sensor, execute measurements, and for analog-to-digital conversion and data transfer. This rough estimates for the supply of the sensor and the amplifier stage are worst-case assumptions and are expected to be even lower in the application. During operation, the system voltage must not drop below 1.8 V, which is required to supply the electronic components. The energy-harvesting capabilities of the system can be validated by connecting the energy-harvesting pin provided by the IC to different load resistors while measuring voltage. Additionally, the distance between sensor-node antenna and reader antenna was changed.

The measurement for the antenna mounted on a ferrite sheet on the multidirectional CFRP plate showed that energy-harvesting capability is currently limited to 11 mW with a maximal distance between sensor-node antenna and reader antenna of 10.8 mm. Reducing the power demand to 5.7 mW allows for operating the system at larger distances of up to 18 mm. The achieved distance increases even more in the course of further investigation because the shape of the rectangular reader antenna is not yet optimally matched to the circular sensor node antenna.

## 4. Conclusions and Outlook

Results presented in this paper clearly demonstrate the validity of the new concept of using the outer stainless-steel layer of an FML as an antenna. Mechanical simulations confirmed that a circular antenna is best suited to be manufactured into the outer layer of a load-bearing structure composed of FML. The use of thin ferrite layers directly underneath the antenna provides two main advantages: electrical insulation and independence from the composite material used in the immediate vicinity of the antenna structure. This approach allows for embedding sensor nodes for SHM into the FML, and for using the existing outer metal layer to create an antenna, eliminating the necessity for external wiring or additional components outside the material itself.

Additionally, results of this paper show that, even for a reader with a geometrically nonoptimized antenna, the harvested energy from the reader field is sufficient to meet the estimated energy requirements of a sensor node with comparably large amounts of required energy due to high analog-to-digital conversion rates. With these findings, it is also possible to provide enough energy for other sensor nodes requiring equal or less energy for multiple other applications.

As the next steps, investigations will be performed regarding the miniaturization of the antenna structure and the effect on its energy-harvesting ability. After that, a demonstration of the antenna integrated into a real FML plate will be performed, and the influence of additional metal layers inside the material stack will be investigated.

## Figures and Tables

**Table 1:** Laser fabrication parameters for cutting groves into 100 μm thick stainless-steel sheet metal.

Laser Parameter	Value	Unit
Wave length	1028	nm
Repetitions	155	
Pulse energy	16	μJ
Pulse duration	236	fs
Mark speed	1200	mm/s
Repetition rate	600	kHz
Laser spot diameter	25	μm

**Table 2:** Layer configurations for plate specimen used in experiments.

Layer No.	UD	MD45	MD90
1	0${}^{\circ }$	+45${}^{\circ }$	0${}^{\circ }$
2	0${}^{\circ }$	−45 ${}^{\circ }$	90${}^{\circ }$
3	0${}^{\circ }$	+45${}^{\circ }$	0${}^{\circ }$
4	0${}^{\circ }$	−45 ${}^{\circ }$	90${}^{\circ }$
5	0${}^{\circ }$	0${}^{\circ }$	0${}^{\circ }$
6	0${}^{\circ }$	0${}^{\circ }$	90${}^{\circ }$
7	0${}^{\circ }$	0${}^{\circ }$	90${}^{\circ }$
8	0${}^{\circ }$	0${}^{\circ }$	0${}^{\circ }$
9	0${}^{\circ }$	−45 ${}^{\circ }$	90${}^{\circ }$
10	0${}^{\circ }$	+45${}^{\circ }$	0${}^{\circ }$
11	0${}^{\circ }$	−45 ${}^{\circ }$	90${}^{\circ }$
12	0${}^{\circ }$	+45${}^{\circ }$	0${}^{\circ }$

**Table 3:** Antenna design parameters.

Antenna Parameter	Value	Unit
Number of turns	8.00	
Inner diameter	24.36	mm
Outer diameter	51.60	mm
Conductor width	1.03	mm
Gap thickness	0.56	mm
Theoretical inductance using Equation (2)	3.01	μH
Theoretical f${}_{res}$ in air (C${}_{tun}$ = 28.5 pF)	17.18	MHz
Desired f${}_{res}$ in application	13.56	MHz

**Table 4:** Power requirements of an SHM sensor node.

Consumer	Power [mW]
Microcontroller STM32L031K6 (sleep)	0.10
Direct memory access	0.58
ADC sampling @1.14 MHz × 3	1.46
RFID IC	0.54
Amplifier and filter (max.)	1.00
Sensor supply (max.)	2.00
Total	5.68

## Data Availability

Data presented in this study are available on request from the corresponding author.

## Abbreviations

The following abbreviations are used in this manuscript:

CFRP	Carbon-fiber-reinforced polymer
FML	Fiber metal laminate
IC	Integrated circuit
RFID	Radio frequency identification
SHM	Structural health monitoring
